# Combined approach (infratentorial supracerebellar–telovelar transventricular) for a large brainstem tumor

**DOI:** 10.3171/2019.10.FocusVid.19409

**Published:** 2019-10-01

**Authors:** Guillermo Aldave

**Affiliations:** Division of Neurosurgery, Department of Surgery, Texas Children’s Hospital; Department of Neurosurgery, Baylor College of Medicine, Houston, Texas

**Keywords:** brainstem tumors, telovelar, supracerebellar approach, video

## Abstract

The safe entry zones into the brainstem provide access to challenging lesions in this region that do not reach the surface. However, in large brainstem tumors there are two issues to bear in mind before deciding the approach. First, the anatomy can be distorted, and it can be difficult to recognize the entry zone. Second, for large brainstem lesions it may be challenging to address the whole tumor from only one zone and combined approaches may be required. Thus, we show a combined approach, infratentorial supracerebellar and telovelar transventricular, to remove a large brainstem tumor. Appropriate consent was obtained.

The video can be found here: https://youtu.be/nCcG9zPq7ug.

## Transcript

**0:20** This is a video to show a combined approach infratentorial supracerebellar and telovelar transventricular for large brainstem tumors.

**0:30** This is the case of a 7-year-old girl with a history of worsening gait due to left leg weakness. The brain MRI showed a midbrain tumor. Stereotactic biopsy and ETV were performed and chemotherapy was started with the diagnosis of pilocytic astrocytoma. Despite treatment the patient presented a worsening of her symptoms. Thus, on her exam she presents a left facial palsy (HB-2), mild left eleventh nerve palsy, left hemiparesis 3/5, reflexes 3/4, gait, unsteady, staggering, ataxic. Only possible with assistance. Left choreic movements.

The follow-up MRI confirmed a significant progression of the tumor, so we decided to pursue surgery for a maximum safe resection.

**1:24** We decide a combined approach for this large brainstem tumor due to the size of the lesion. We are not confident that we could get a good exposure of the whole lesion with only one of the corridors, specially if the tumor does not reach the surface. We are planning to start with supracerebellar infratentorial on the left side, using a lateral corridor, 2 cm far from midline, because it is where the tumor will reach the pia most likely. Therefore, after the midbrain part resection we will continue with the telovelar transventricular approach to access the pons.

**2:00** With the patient with the head flexed and in neutral position over Mayfield headframe, after a suboccipital craniotomy we start the dissection of the supracerebellar fissure on the left side. We expose the cerebellar mesencephalic fissure whose dissection would expose the dorsal aspect of the midbrain. As we move forward with the dissection with microscissors we start seeing the midbrain and a gray lesion that is coming into the surface reaching the pia consistent with the tumor.

**2:30** It is important to spend time releasing the arachnoid of the fissure to facilitate the opening, avoiding excessive retraction over the cerebellum.

**2:41** Having opened the fissure and exposed the tumor, we continue with the coagulation of the pia right over the tumor, leaving the rest of the midbrain intact. The tumor is gray, mildly vascular, and soft in consistency, which allows us an efficient decompression with regular suction or ultrasonic aspirator.

**3:15** As we move forward with the debulking, we start to see the borders or the transition to a more infiltrative part, browner and more yellowish, that we decide to keep to avoid any additional deficits.

**3:30** Having finished with the midbrain component through the supracerebellar approach, we direct our attention to the component in the pons through the transventricular telovelar approach. We dissect the tonsillovermian fissure first to expose the tela and the choroid plexus.

**3:47** We decide to open the tela only in the right side where the tumor could reach the floor of the ventricle. The opening of the tela allows an exposure of three-quarters of the floor of the III. Thus, we identify the stria medullaris bilaterally and the median sulcus. The stria points the infrafacial triangle, but we considered that the suprafacial triangle would be a more accurate entry site, in case the tumor did not reach the surface.

**4:14** Because of the mass effect of the lesion it is tricky to identify the facial colliculus, so we decide not to stimulate it. Besides, there is an area slightly more bluish where we can intuit the tumor. We use it as the entrance into the pons with dissection first and then with bipolar. We are performing the surgery with continuous neuromonitoring of MEPs, SSEPs, brainstem auditory evoked responses (BAERs), and facial EMG.

**4:18** As with the midbrain portion, we identify a gray and soft tumor that can be aspirate with regular suction or ultrasonic aspirator. Specially in the brainstem and during the debulking we do not pursue the borders of the tumor, but we let them come to the center of the surgical field. We use the bipolar not only for hemostasis but mainly to keep the work window open.

**5:15** This is a nice view where we can distinguish the tumor gray from a more normal-looking wall that we leave intact.

**5:36** As it happened in the order approach, we decide to leave the more brown-yellowish–looking tissue that could correspond to infiltrative tumor or just edema.

**5:48** Final view of cerebellum after the resection.

**5:52** This is the postoperative MRI that shows a near-total resection of the contrast enhancement of the tumor. Patient did well after surgery without additional deficits. She was discharged to the rehabilitation facility within 7 days after the procedure. On exam the diplopia and the facial palsy resolved within the first 2 weeks. The choreic movements improved and were better controlled with medication. She was able to stand up, to talk, and to eat on her own. The pathology came back as pilocytic astrocytoma.
